# Synthesis, Characterization, Photo/Electrocatalytic Activity, and Corrosion Behavior of Ag_2_S/PANI Composite Layered TiO_2_ Nanotubes Coated on Ti Foil

**DOI:** 10.1002/gch2.202500011

**Published:** 2025-05-06

**Authors:** Derya Birhan, Derya Tekin, Burak Dikici, Taner Tekin, Hakan Kızıltaş

**Affiliations:** ^1^ Department of Metallurgy and Materials Engineering Ataturk University Erzurum 25240 Turkey; ^2^ Department of Mechanical Engineering Ataturk University Erzurum 25240 Türkiye; ^3^ Department of Chemical Engineering Ataturk University Erzurum 25240 Turkey

**Keywords:** Ag_2_S NPs, anodization, conductive polymers, corrosion, photo/electrocatalytic, TiO_2_ nanotube

## Abstract

In this study, the photo/electrocatalytic and electrochemical properties of the composite structure formed by coating silver sulfide (Ag_2_S) and polyaniline (PANI) on titanium nanotubes are investigated. Titanium nanotubes (TiO_2_ NTs) are synthesized on the surface of titanium (Ti) sheets using the anodization method. Ag_2_S is deposited on the surface of TiO_2_ NTs through the successive ionic layer adsorption and reaction (SILAR) method, while PANI is coated via the chemical oxidative polymerization technique. SEM‐EDS, XRD, FTIR, and electrochemical measurements are used to characterize the prepared nanotubes. The corrosion inhibitor performance of TiO_2_ NTs is investigated by electrochemical impedance spectroscopy (EIS) and potentiodynamic polarization (PDS) measurements. EIS measurements showed that the PANI layer decreased the resistance of the photoanodes, while the Ag_2_S coating increased the resistance compared to PANI. It is determined that the semicircles in the Nyquist curves drawn using EIS measurements are formed due to the Warburg resistance in the low‐frequency region and the high‐frequency region caused by the charge transfer resistance. Corrosion parameters are measured using Tafel fitting curves. Overall, TiO_2_ NTs grown by anodization effectively prevented the corrosion of the Ti photoelectrode, and the corrosion rate is calculated at 0.013 mm/year (mpy). In electrocatalytic (EC), photocatalytic (PC), and photoelectrocatalytic (PEC) activity experiments, PANI/Ag_2_S/TiO_2_ NT photoelectrode removed 13.775%, 37.71%, and 61.075% of methylene blue (MB) within 120 min under UV light irradiation.

## Graphical abstract

This study explores the corrosion resistance and photoelectrocatalytic performance of TiO_2_ nanotubes (TiO_2_ NTs) coated with silver sulfide (Ag_2_S) and polyaniline (PANI). Electrochemical impedance spectroscopy (EIS) and potentiodynamic polarization techniques are used to evaluate the corrosion behavior of the samples in a simulated corrosive environment. The synergistic effect of Ag_2_S and PANI coatings significantly enhanced the corrosion resistance compared to bare TiO_2_ NTs. Additionally, the photoelectrocatalytic activity is analyzed under simulated solar illumination, demonstrating improved charge separation and photocurrent response. The results indicate that Ag_2_S/PANI‐modified TiO_2_ NTs exhibit superior anti‐corrosion and photoelectrocatalytic properties, making them promising candidates for sustainable energy and protective coating applications.

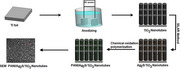

## 1 Introduction

With the emergence of green electricity production in recent years, electrochemical technologies are gaining importance. Hybrid processes developed by combining electrochemical steps with biological, chemical, or physical methods are now widely used. As an example of these hybrid processes, we can give the photoelectrocatalytic (PEC) process, which occurs by combining electrochemical and photochemical events. The emergence of the PEC process is important for photocatalytic (PC) activity and increases PC activity.^[^ ^1^ ^]^ The use of photocatalytic technology to solve environmental pollution and energy crises is developing thanks to its high efficiency features, and clean and economical features. By this time, visible and UV light‐sensitive materials have been widely studied and excellent materials have been developed.^[^ ^2^ ^]^ Titanium dioxide (TiO_2_) is a typical semiconductor used for reasons such as low cost, high stability, wide band gap, nontoxic, and high quantum efficiency of light‐induced charge.^[^ ^3^ ^]^ However, the charge separation rate and low quantum efficiency of TiO_2_ greatly limit its photocatalytic activity.^[^ ^4^ ^]^ Recently, various forms of TiO_2_, including hollow spheres,^[^ ^5^ ^]^ nanowires,^[^ ^6^ ^]^ nanoparticles,^[^ ^7^ ^]^ nanosheets,^[^ ^8^ ^]^ and nanotubes,^[^ ^9^ ^]^ have been developed to solve the above‐mentioned problems. In particular, ordered structures of TiO_2_, such as nanotubes, exhibit a higher surface area/volume ratio compared to other forms. Among these forms, TiO_2_ nanotubes^10^ ^]^ are unique as they have excellent properties such as large specific surface area, short electron transfer paths, highly vertically oriented ordered structures, and charge transfer channels.^[^ ^11^, ^12^, ^13^ ^]^ Although the morphology of TiO_2_ NTs has expanded the light absorption regions and increased the number of reaction sites of the photocatalyst, this structure alone does not satisfactorily improve the photocatalytic performance of TiO_2_. A high surface area/volume ratio reduces the recombination rate by increasing charge separation efficiency and electron transport rate. Additionally, these materials make UV light or sunlight ideal for photoelectronic applications. These features offer a wide range of applications in many technological fields such as solar cells, battery electrodes, and sensors. The most important of these areas are photocatalytic (PC) and photoelectrocatalytic (PEC) application areas.^[^ ^1^, ^14^ ^]^ TiO_2_ used in PEC activity is the most preferred photocatalyst in PEC separations due to reasons such as being environmentally friendly, photoactive, resistant to chemical corrosion and photo corrosion, and economical. However, TiO_2_ wide band gap of 3.2 eV absorbs only 4% of solar energy and releases 48%. In recent years, to increase photocatalytic activity, sulfide‐based materials with narrow band gaps (MoS_2_, CuS, NiS, CdS, SnS_2_, Bi_2_S_3_, and Ag_2_S)^[^ ^15^ ^]^ have been doped. Among these materials, silver sulfide (Ag_2_S) is a direct band gap (0.9–1.05 eV)^[^ ^16^ ^]^ highly stable semiconductor, has superior catalytic potential, is nontoxic, has unique electrical and optical properties, is economical, and has antibacterial properties.^[^ ^17^, ^18^ ^]^

Polyaniline (PANI) is the most extensively researched type of conductive polymer due to its environmental stability, easy synthesis, unique reversible protonic capability, variable electrical conductivity, and excellent redox reversibility.^[^ ^19^, ^20^ ^]^ It is possible to synthesize PANI through chemical/electrochemical, acid/base treatment, and oxidation/reduction. K. Lim et al synthesized conductive PANI/TiO_2_ nanotube rods using chemical oxidation polymerization.^[^ ^21^ ^]^ Although there are studies on metal sulfides and conductive polymers, photoelectrodes with Ag_2_S and PANI coated on nanotubes grown on TiO_2_ foil are unavailable in the literature. Chen et al, who synthesized PANI, an excellent conductive polymer, through chemical oxidation, successfully synthesized the CuS/PANI composite structure with a core‐shell structure.^[^ ^22^ ^]^ In this study, they achieved a specific capacitance of 308.1 F g^−1^ at 0.5 A g^−1^ of this composite structure. In addition, PANI photoelectrodes attract attention for reasons such as being easy to synthesize, environmentally stable, good electrical conductivity, and economical. They are used to make TiO_2_ nanotubes photosensitive to significantly increase the photocatalytic activity efficiency under visible light due to their delocalized conjugated structure during electron transfer.^[^ ^23^ ^]^ The PANI‐modified metal sulfide structure was synthesized by Xiu Fang Wang et al.^[^ ^24^ ^]^ This synthesized structure was used in the photocatalytic degradation of Rhodamine B dye and it was determined that more than 90% of the dye was removed.

In this study, TiO_2_ nanotube photoelectrodes were synthesized using the anodization method, and Ag_2_S/TiO_2_ nanotubes were synthesized using the SILAR method. By taking advantage of the strong interaction of Ag_2_S/TiO_2_ nanotubes, the photoelectrocatalytic activity of photoelectrodes can be improved, so that Ag_2_S/TiO_2_ NTs can be efficiently used in the removal of methylene blue. Finally, polyaniline synthesized by the chemical oxidation polymerization method was coated on the surface of Ag_2_S/TiO_2_ NTs, and the electrochemical properties of the nanotubes were examined. Characterization of PANI/Ag_2_S/TiO_2_ NT photoelectrodes was performed by SEM‐EDS, XRD, and FTIR analyses. Electrochemical measurements were made using a three‐electrode system in 1M H_2_SO_4_ solution, and the photoelectrocatalytic activities of PANI/Ag_2_S/TiO_2_ NT photoelectrodes were examined using methylene blue dye.

## 2 Results and Discussion

### 2.1 SEM‐EDS Analyzes

**Figure 1:**
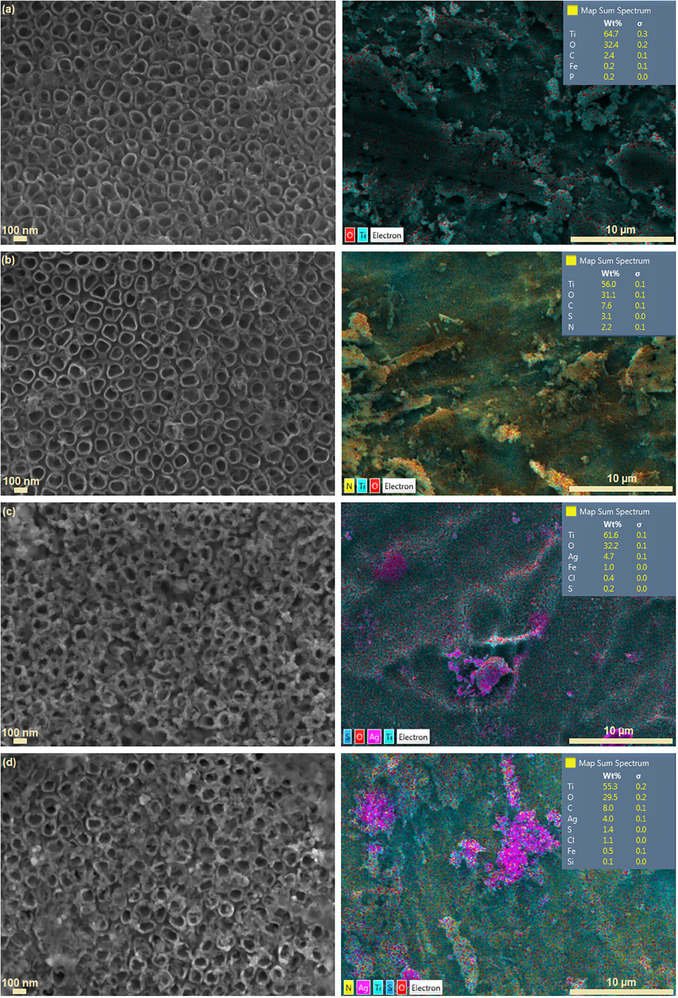
SEM‐EDS mapping images of a) TiO_2_ NT, b) PANI/TiO_2_ NT, c) Ag_2_S/TiO_2_ NT and d) PANI/Ag_2_S/TiO_2_ NTs.

**Figure** **1** shows SEM‐EDS images of TiO_2_, PANI/TiO_2_, Ag_2_S/TiO_2_ and PANI/Ag_2_S/TiO_2_ NTs. In the SEM image of TiO_2_ NTs in Figure 1a, it is seen that the nanotubes synthesized by anodization are self‐organized and vertically oriented. The O/Ti ratio of TiO_2_ NTs was calculated as ≈1.49. The images show that the nanotube arrays are tightly aligned, and the average nanotube diameter is 100 nm.

In the SEM image of PANI/TiO_2_ NTs shown in Figure 1b, it is seen that the nanotubes prepared by the anodization process at 20 V for 1 h under optimized conditions have a highly ordered structure and the average diameter is 100 nm. PANI solution with a concentration of 0.01 m coated on the surface of TiO_2_ NTs adheres to the surface of the nanotubes like a gel, and the diameter of the nanotubes is observed to decrease.

Figure 1c shows the SEM image of Ag_2_S/TiO_2_ NT. It is seen that Ag_2_S nanoparticles coated using the SILAR method have a relatively uniform distribution on the surface of TiO_2_ NTs. After five repeated SILAR cycles, it is understood from the SEM images that Ag_2_S nanoparticles densely accumulate around the tubes.^[^ ^25^ ^]^ The average diameter of Ag_2_S nanoparticles coated on TiO_2_ nanotubes by the SILAR method is between 10 and 25 nm.

Figure 1d shows the SEM image of PANI/Ag_2_S/TiO_2_ NTs. PANI synthesized by chemical oxidation polymerization method was coated on the Ag_2_S/TiO_2_ NTs surface like a gel. From the SEM image, it can be seen that Ag_2_S nanoparticles adhere to the walls of TiO_2_ NTs and PANI covers this structure. It can be seen that the diameters of the nanotubes are larger than those of Ag_2_S/TiO_2_ NTs. The reason for this is that the agglomeration of Ag_2_S decreases as a result of the electrostatic interaction between Ag_2_S nanoparticles and PANI due to the positive charge of PANI. Although some tubes are seen to be closed due to doping, TiO_2_ nanotubes synthesized by the anodization method have a size of 100 nm, and it is understood from the SEM images that the doped Ag_2_S nanoparticles and PANI coating cause the thickness of the nanotube walls to increase.

EDS mapping was performed and the results are shown in Figure 1. The presence of N, O, Ti, S, and Ag elements confirmed the formation of the PANI/Ag_2_S/TiO_2_ NT structure. EDS analysis showed that all elements in the structure were well distributed.

### 2.2 XRD Analysis

**Figure 2:**
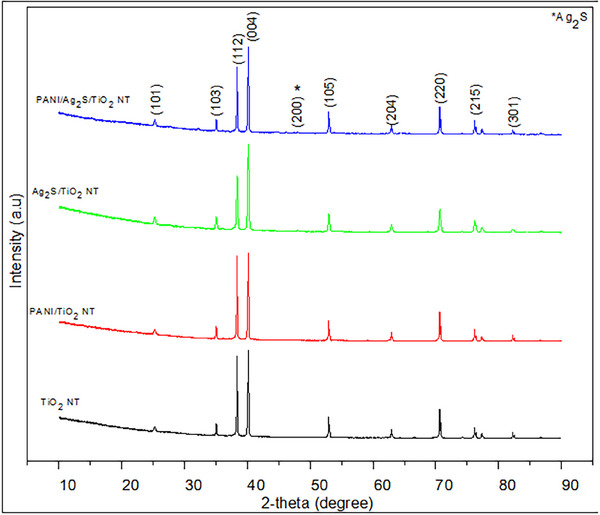
XRD diagram of TiO_2_, PANI/TiO_2_, Ag_2_S/TiO_2_, and PANI/Ag_2_S/TiO_2_ NTs.

The crystal lattice structures of TiO_2_, PANI/TiO_2_, Ag_2_S/TiO_2,_ and PANI/Ag_2_S/TiO_2_ NTs were examined by XRD in the diffraction peaks seen in **Figure** **2**.

The 2θ peaks of TNTs at 25.27°, 35.14°, 38.34°, 40.16°, 53.11°, 55.07°, 62.96°, 70.61°, 76.23°, and 82.41° correspond to the (101), (103), (112), (004), (105), (105), (211), and (201) planes. The diffraction peaks of TiO_2_ NTs (JCPDS No. 21‐1272) are in perfect harmony with the cards. At the 2θ peaks, diffraction peaks belonging to Ag_2_S nanoparticles were seen and were compatible with (JCPDS No 14‐0072) cards.^[^ ^26^ ^]^ The small peak seen at 48.07 in the XRD diagram of Ag_2_S/TiO_2_ and PANI/Ag_2_S/TiO_2_ NT corresponds to the (200) plane and it is understood that this peak belongs to Ag_2_S. Ag_2_S nanoparticles with monoclinic structure show diffraction peaks in the same places as TiO_2_. No obvious diffraction peak exists in samples containing sulfur due to its small size and amount.^[^ ^27^ ^]^ It is understood that the peaks belonging to the anatase TiO_2_ phase are found in a dispersed form of recrystallized Ag_2_S nanoparticles at the observed angles. The small peak seen at 48.07 belongs to Ag_2_S and is seen in the XRD diagram. The Ag_2_S content in the Ag_2_S/TiO_2_ and PANI/Ag_2_S/TiO_2_ composite structures is 4.33% and 4.05% by weight. The small peak seen in the range of 30°‐35° in the XRD graph of PANI/Ag_2_S/TiO_2_ NT is also observed in the Ag_2_S/TiO_2_ NT graph. The peak is a reflection of the Ag_2_S crystal phase. It is thought that the PANI coating causes the intensity of the XRD peak to increase since it stabilizes the crystal structure of Ag_2_S.

The reaction mechanism of metallic Ag formation is as follows.

(1) $$\mathrm{AgN}O_{3}\to Ag^{+}+{\mathrm{NO}}_{3}^{-}$$

(2) $$Na_{2}S\to 2N_{2}^{+}+S^{-2}$$

(3) $$2Ag^{+}+\;\;S^{-2}\to Ag_{2}S$$

Metallic Ag and SO_2_ are produced during the calcination process conducted at 500 °C.

(4) $$2Ag_{2}S+3O_{2}\to 4\mathrm{Ag}+2SO_{2}$$

### 2.3 FTIR analysis

**Figure 3:**
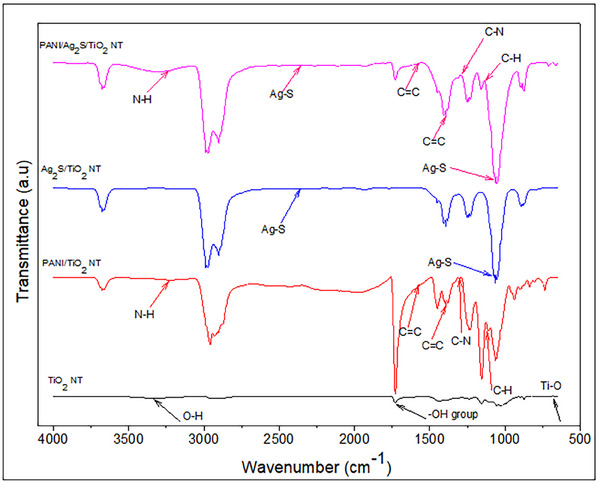
FTIR spectrum of TiO_2_, PANI/TiO_2_, Ag_2_S/TiO_2_ and PANI/Ag_2_S/TiO_2_ NTs.

The FTIR spectra of TiO_2_, PANI/TiO_2_, Ag_2_S/TiO_2_ and PANI/Ag_2_S/TiO_2_ NTs are shown in **Figure** **3**. In the FTIR spectrum of TiO_2_ nanotubes, the band seen at 3343 cm^−1^ corresponds to O‐H stretching vibration, while the band seen at 1725 cm^−1^ is attributed to the presence of hydroxyl groups (─OH). The band at 683 cm^−1^ corresponds to Ti─O─Ti stretching vibrations. The peak seen at 2360 cm^−1^ is due to the Ag─S bonds in the structure of Ag_2_S. PANI/Ag_2_S/TiO_2_ and Ag_2_S/TiO_2_ NTs show the absorption band of Ag‐S ≈1062 cm^−1^.^[^ ^28^ ^]^ The low prominence of the peak at 2360 cm^−1^ is because the denser the Ag_2_S coating, the lower the density of functional groups containing the peak. The chemical bond structure of TiO_2_, PANI/TiO_2_, Ag_2_S/TiO_2_, and PANI/Ag_2_S/TiO_2_ NTs was confirmed by FTIR analysis. In the FTIR spectrum given in Figure 3, the peak of PANI/TiO_2_ and PANI/Ag_2_S/TiO_2_ NTs at 3236 cm^−1^ corresponds to the symmetric N─H stretching band. The absorption peaks at 1401 and 1575 cm^−1^ correspond to the C=C bond formed by aromatic ring stretching vibrations of quinoid and benzenoid rings. While the low‐frequency bands at 1297 cm^−1^ correspond to C‐N vibration, the peak at 1136 cm^−1^ corresponds to C─H in‐plane bending, and the peaks at 825 and 504 cm^−1^ correspond to C─H out‐of‐plane bending.^[^ ^29^ ^]^

### 2.4 Photocatalytic (PC), Electrocatalytic (EC) and Photoelectrocatalytic (PEC) Activity Properties of TiO_2_ NT Photoelectrodes

**Figure 4:**
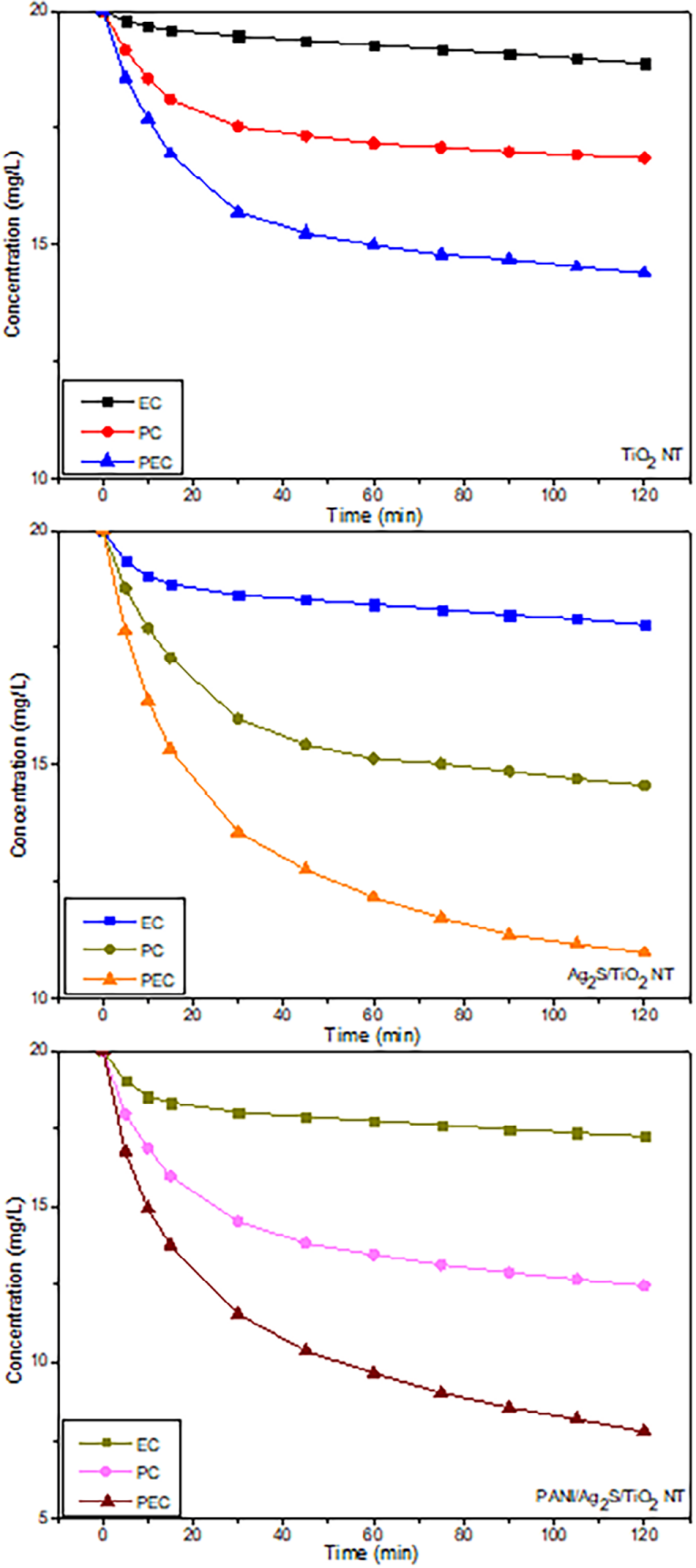
Electrocatalytic, photocatalytic and photoelectrocatalytic degradation graphs of TiO_2_, Ag_2_S/TiO_2_ and PANI/Ag_2_S/TiO_2_ NT photoelectrodes on methylene blue dye.

Photocatalytic, electrocatalytic, and photoelectrocatalytic experiments of TiO_2_, Ag_2_S/TiO_2,_ and PANI/Ag_2_S/TiO_2_ NTs lasted 120 min and the results are shown in **Figure** **4**.

The dye removal efficiency of TiO_2_ NTs was evaluated by examining their degradation in methylene blue aqueous solution. PEC activity of TiO_2_ photoelectrodes prepared at 20V anodization voltage and 500 °C annealing temperature was evaluated in MB solution prepared with an initial dye concentration of 20 ppm containing 0.05m Na_2_SO_4_. Studies in the literature have determined that nanotube arrays created by anodization at 20V lead to high PEC activity.^[^ ^30^, ^31^ ^]^ TiO_2_ nanotubes removed the MB solution by photocatalytically 15.74%, electrocatalytically 5.635%, and photoelectrocatalytically 28.02% of the MB solution within 120 min. The photocatalytic performance of Ag_2_S/TiO_2_ NTs on MB was higher than that of pure TiO_2_, and it removed 27.215% of the dye within 120 min. Ag_2_S/TiO_2_ NT shows better performance than pure TiO_2_. The main reason is that Ag_2_S nanoparticles expand the response range of TiO_2_ to light and reduce the recombination rate of photoinduced electron‐hole pairs. PC, EC, and PEC removal graphs of the synthesized samples are shown in Figure 4. In EC and PEC processes using Ag_2_S/TiO_2_ NT photoelectrode under UV irradiation of MB, 10.075% and 45.09% removal were achieved, respectively. Accordingly, since the EC removal of MB without UV light irradiation is less than the PEC and PC removals, it is understood that the degradation of MB into EC cannot be done quickly.^[^ ^32^ ^]^ As a result of the bias potential of 0.6 V applied to Ag_2_S/TiO_2_ NTs, the PEC decay rate of MB is ≈1.6 times that of the PC degrade rate. It was determined that the PC and PEC decay rates are 1.7 and 1.6 times higher than pure TiO_2_ NTs, respectively. Ag_2_S/TiO_2_ NTs have the second‐best removal performance in terms of PEC. This is because the applied bias potential prevents the recombination of photogenerated electron‐hole pairs, thus extending the lifetime of photogenerated carriers. The EC, PC and PEC degradations of PANI/Ag_2_S/TiO_2_ NT photoelectrode on methylene blue are shown in Figure 4. As shown in Figure 4, PANI/Ag_2_S/TiO_2_ NT removed 37.71% of methylene blue as PC. In the EC experiment where 0.6V voltage was applied, it removed 13.775% of the dye and in the PEC experiment, it removed 61.075% of the dye. The PEC degradation activity of MB is higher than the other samples, 2.18 times higher than TiO_2_ NT and 1.35 times higher than Ag_2_S/TiO_2_ NT.

PANI/Ag_2_S/TiO_2_ NTs showed different degradation efficiencies of 13.78%, 37.71%, and 61.075% in EC, PC, and PEC activities for 2 h, respectively. When compared with pure TiO_2_ and Ag_2_S/TiO_2_ NTs, PC and PEC activities are pretty good except for EC degradation rates. It is understood from the degradation experiments that the modification of PANI can prevent the recombination of electron‐hole pairs of TiO_2_ NTs under UV light and thus increase the degradation performance of organic dyes.^[^ ^33^ ^]^

### 2.5 Electrochemical Impedance Spectroscopy (EIS) of TiO_2_ NT Photoelectrodes

**Figure 5:**
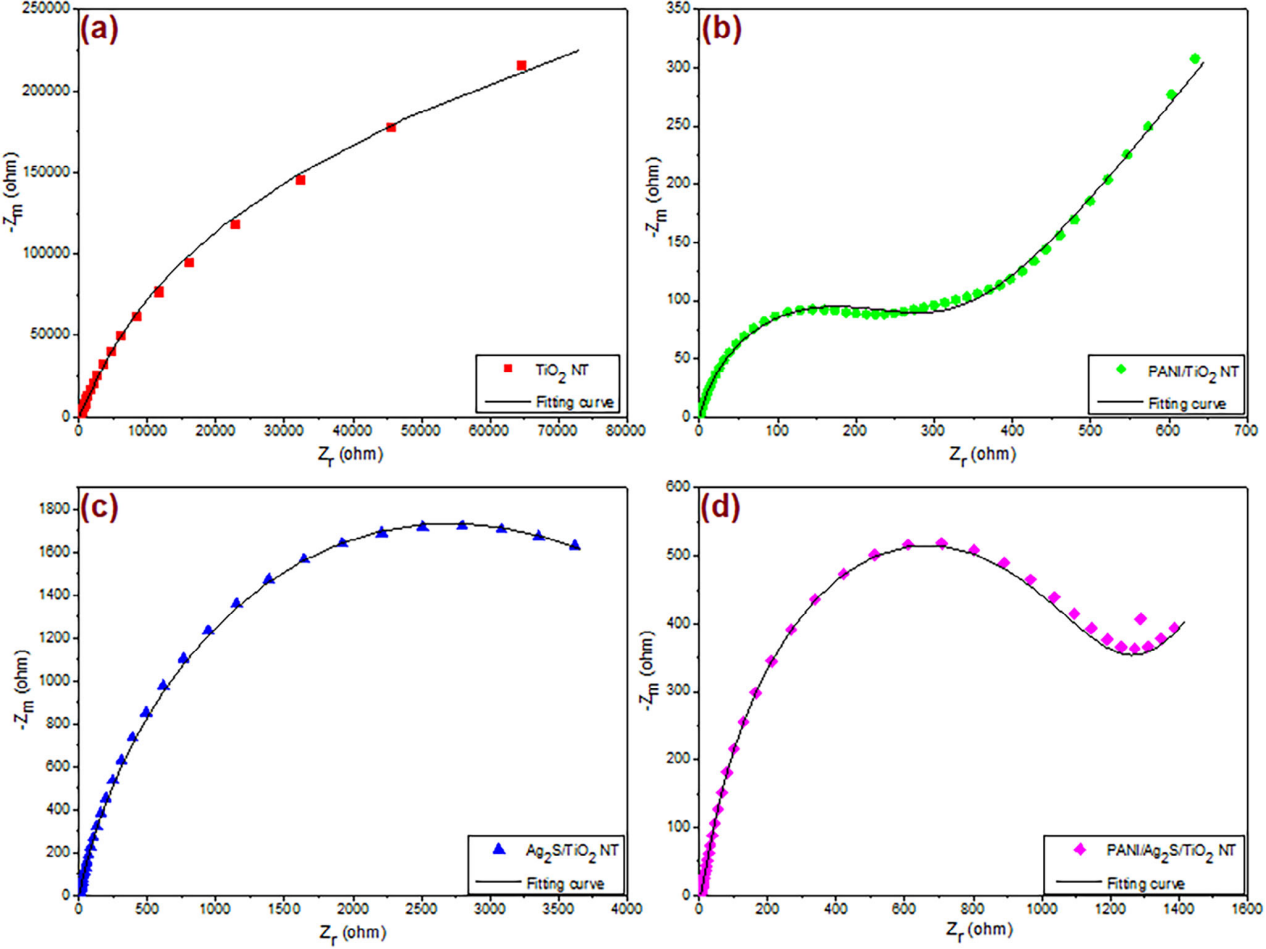
Nyquist plots of a) TiO_2_, b) PANI/TiO_2_, c) Ag_2_S/TiO_2_ and d) PANI/Ag_2_S/TiO_2_ NTs in 1M H_2_SO_4_ solution.

**Figure 6:**
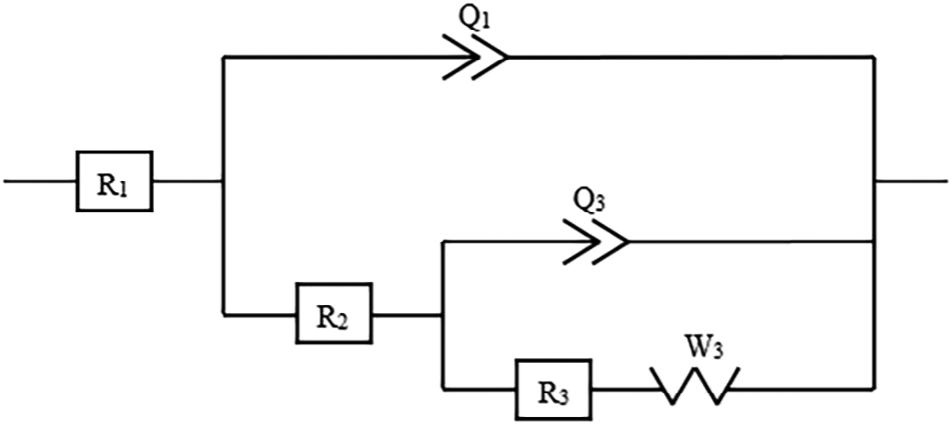
An equivalent electrical circuit model used to fit all experimental impedance data.

Nyquist plots are shown in **Figure** **5** after taking open circuit potential (OCP) for 2 h in 1 m H_2_SO_4_ solution. According to electrochemical theory, a current diagram is obtained by charging and discharging the double layer at the interface and oxidation/reduction of chemicals on the electrode surface or in solution. The equivalent electrical simple model circuit for EIS measurements is also shown in Figure 5. The appropriate equivalent circuit for Nquist plots was determined as R1 + Q1/R2 + Q2/(R3 + W3). Nyquist plots and the corresponding equivalent circuit connection of PANI and Ag_2_S‐doped TiO_2_ nanotube photoelectrodes are shown in Figure 5a–d. In the impedance and corrosion tests performed on TiO_2_, PANI/TiO_2_, Ag_2_S/TiO_2_, and PANI/Ag_2_S/TiO_2_ NT samples, 3 repetitions were made to ensure that the results were fully compatible with each other. **Figure** **6** shows the equivalent circuit model suitable for impedance data suitable for all photoelectrodes. **Table** **1** shows the EIS results performed in 1m H_2_SO_4_ solution.

**Table 1:** EIS analysis results obtained in 1 m H_2_SO_4_ solution.

Samples	R_1_ [Ω]	Q_1_	R_2_[Ω]	Q_2_	R_3_[Ω)]	W_3_
TiO_2_ NT	0.1461	24.27e‐6	11.09	36.25e‐6	2.437e6	2796
PANI/TiO_2_ NT	0.4048	0.8039e‐3	0.125	72.73e‐6	467.8	104.2
Ag_2_S/TiO_2_ NT	0.1903	0.5302e‐3	102.6	0.248e‐3	3916	181.7
PANI/Ag_2_S/TiO_2_ NT	1.254	0.2324e‐3	6.259	0.3824e‐3	1323	52.86

In EIS analysis, if we need to explain each component in the R1 + Q1/R2 + Q2/(R3 + W3) equivalent circuit model used, R1: Represents the resistance due to ionic conductivity at the electrode‐electrolyte interface or in the solution. Q1 (Constant Phase Element‐CPE1): Used to model non‐ideal double‐layer capacitance. R2 (Interfacial Resistance or Polarization Resistance): Represents the charge transfer resistance between the electrode and the electrolyte. It is related to corrosion or electrochemical reaction processes. Q2 (Constant Phase Element‐CPE2) is used to represent the deviation from the second capacitance, while R3 (Diffusion Resistance or Film Resistance) represents the resistance of the passive film, thin layer, or reactive interface formed on the electrode surface. W_3_ (Warburg Impedance‐Diffusion Impedance): Describes diffusion processes due to mass transport. Table 1 shows that the series solution resistance (R1) increases with PANI doping but decreases with Ag_2_S doping. It is observed that the PANI doping of 0.01 m increases the solution resistance, which is responsible for enhancing the electrochemical supercapacitive performance of photoelectrodes. The impedance graph should be vertical and parallel to the imaginary impedance axis for an ideal photoelectrode. The charge transfer between the electrode and the electrolyte is represented by the semicircular arcs indicated by the electrode material in the Nyquist plots shown in Figure 5a–d. The semicircles here show that the system has an electrochemical interface. Warburg impedance (W_3_) refers to a process controlled by diffusion in the lower frequency range, that is, in a linear part. A smaller charge transfer resistance and a higher charge separation efficiency are represented by a smaller semicircle. The Nyquist curves shown in Figure 5 consist of a linear section in the low‐frequency region resulting from the Warburg resistance and a semicircular section in the high‐frequency region related to the charge transfer resistance. In comparative EIS studies of the samples, the semicircles of TiO_2_ NTs^[^ ^34^ ^]^ are larger than those of other samples.^[^ ^35^ ^]^ This shows that the resistance of the TiO_2_ sample is higher than other photoanodes. As the Ag_2_S film is coated on the surface, the resistance of the photoanode decreases. The coating polymer layer on the nanotube significantly reduces the resistance of the photoanode. It is understood that the PANI/Ag_2_S/TiO_2_ NT photoanode shows more resistance than the PANI/TiO_2_ NT. This shows that PANI reduces the resistance of photoanodes more than Ag_2_S. After the weight measurements were made after the coating process, the amount of Ag_2_S coated on the Ag_2_S/TiO_2_ NT surface is 0.03 g, and the PANI and Ag_2_S coating on the PANI/Ag_2_S/TiO_2_ NT surface is 0.09 g. In this study, the R2 value (69.93 Ω) of Ag_2_S/TiO_2_ NT may have shown a high value because Ag_2_S is a semiconductor and partially prevents charge transfer. This indicates that electron transitions are difficult at the interface and increase recombination. PANI coating on Ag_2_S/TiO_2_ NT significantly reduced R2 and improved charge transportability. The fact that the PANI/Ag_2_S/TiO_2_ NT (6.259 Ω) sample has the lowest W3 value indicates that ions are transported very quickly. Therefore, Ag_2_S/TiO_2_ NTs coated with PANI are the samples with the best electrochemical performance.

### 2.6 Potentiodynamic Polarization Study (PDS) of TiO_2_ Nanotubes

**Figure 7:**
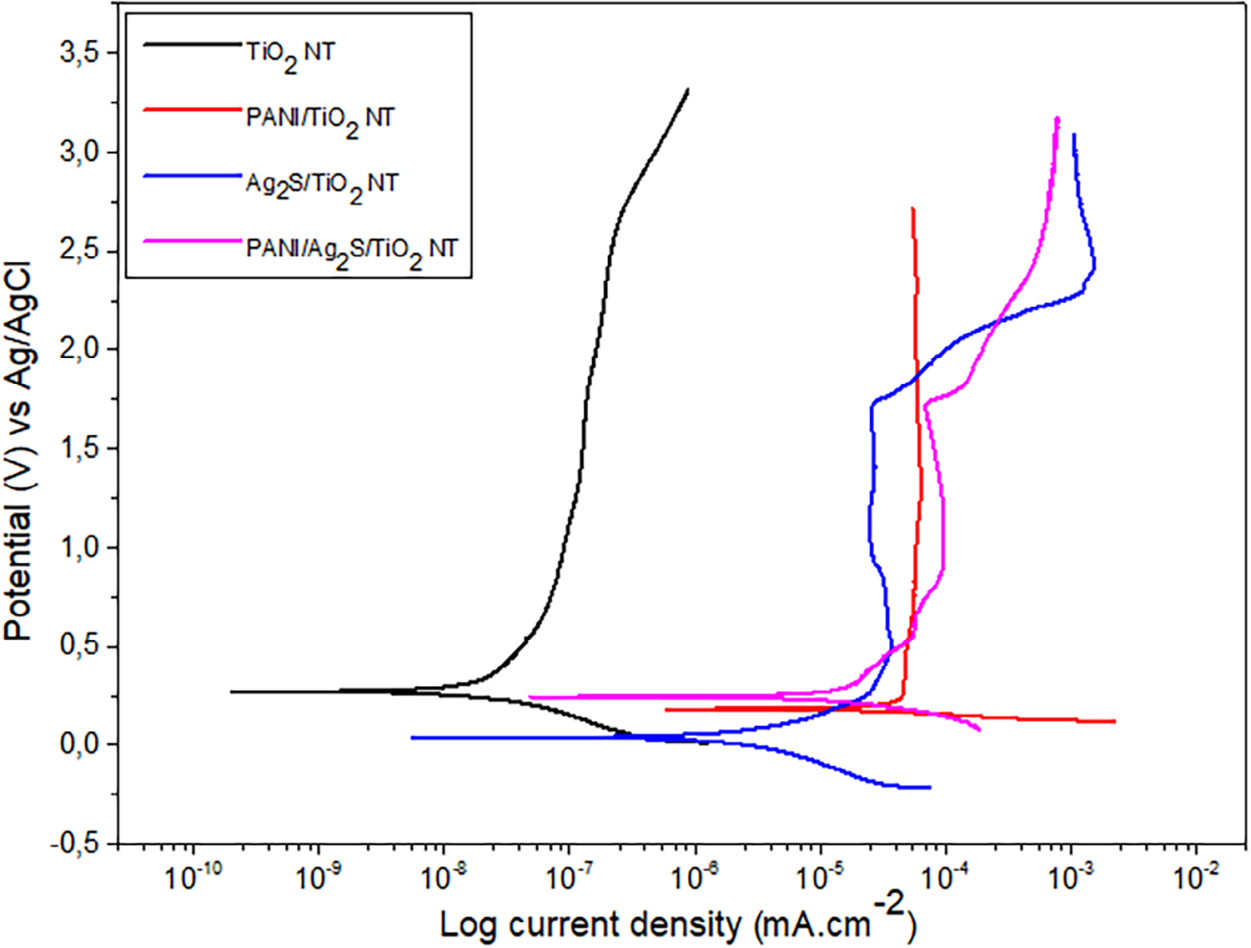
Polarization curves of TiO_2_ NTs coated with 0.01 m Ag_2_S and PANI in 1M H_2_SO_4_ solution.

The potentiodynamic polarization study performed in 1M H_2_SO_4_ solution for 0.01 m Ag_2_S and PANI‐coated samples is shown in **Figure** **7**. Calculated polarization parameters for TiO_2_ NTs are given in **Table** **2**. The Icorr and Ecorr values ​​of each sample were calculated based on Tafel extrapolation and ranged from 0.046 to 166.802 µA cm^−2^, and 33.003 to 302.355 mV, respectively. TiO_2_ and Ag_2_S/TiO_2_ NTs showed good corrosion resistance compared to other samples.

**Table 2:** The average value of polarization parameters for TiO_2_ NTs coated with 0.01 m Ag_2_S and PANI and anodized at 20 V.

Samples	E_corr_ [mV]	i_corr_ [µAcm^−2^]	Β_a_ [mV dec^−1^]	Β_c_ [mV dec^−1^]
TiO_2_ NT	302.355	0.046	562.4	210.2
PANI/TiO_2_ NT	161.859	116.802	9586.7	27.1
Ag_2_S/TiO_2_ NT	33.003	7.546	227.5	221.6
PANI/Ag_2_S/TiO_2_ NT	294.229	48.632	585.4	199.0

It was observed that due to the formation of a stable oxide layer at a potential value of >0.5 V, no further corrosion was observed in the samples and a passive zone that was highly effective against corrosion was formed. No further corrosion occurred due to this passive zone. Ag_2_S/TiO_2_ NT and PANI/Ag_2_S/TiO_2_ NT formed a passive layer that fractured and reformed in a three‐stage process. Coating the TiO_2_ NT surface with Ag_2_S caused an increase in corrosion potential and a decrease in corrosion current density. Ag_2_S reduced the corrosion rate by forming a protective barrier on the surface. However, the Ag_2_S coating alone cannot reach the superior protection performance provided by the double‐layer coating used with PANI. Samples coated with PANI and Ag_2_S showed the lowest corrosion current and the highest corrosion potential. PANI, which has conductive properties, contributes to forming an effective passive layer when combined with Ag_2_S, making the TiO_2_ surface resistant to corrosion.

**Figure 8:**
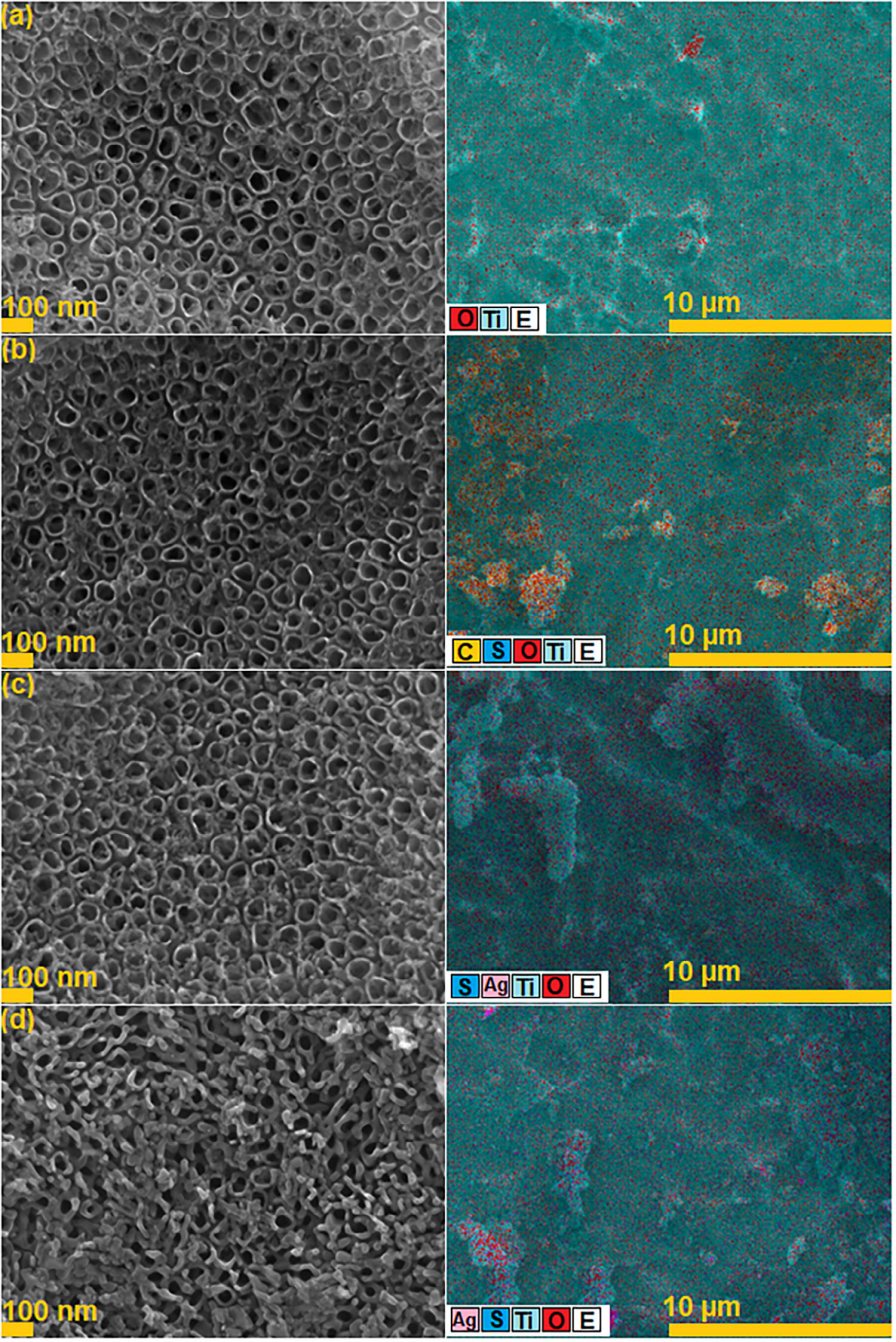
SEM‐EDS images of a) TiO_2_, b) PANI/TiO_2_, c) Ag_2_S/TiO_2_ and d) PANI/Ag_2_S/TiO_2_ NTs after corrosion tests in 1 m H_2_SO_4_.

**Figure** **8** shows the corroded images of pure, Ag_2_S, and PANI‐coated NTs. It is seen that especially corrosion marks on the surface of PANI/TiO_2_ NT are formed more and the surface is destroyed. For Ag_2_S/TiO_2_ NT, it is seen that the pores are opened and the Ag nanoparticles are corroded. As seen from the EDS images, a very dense oxide layer has formed on the structures.

In future studies, it is possible to change parameters such as anodization time, pH ratios, and metal doping amounts or to conduct studies in nanocomposite form. It is also used in supercapacitor studies due to PANI's electrical conductivity.

## 3 Conclusion

This study includes the easy production process of PANI/TiO_2_ NTs produced by chemical oxidation polymerization synthesis of Ag_2_S‐doped TiO_2_ NTs and aniline monomer. Characterization was performed using SEM‐EDS, XRD, and FTIR devices. Electrochemical tests were carried out in 1 m H_2_SO_4_ solution using a potentiostat. The results show that the nanotubes are composed of TiO_2_ NTs doped with Ag_2_S and PANI in the anatase phase. SEM results show that the nanotube arrays formed by anodization are quite regular and homogeneously distributed oxide layers are formed on them. XRD results showed that the produced TiO_2_ NTs were compatible with all peaks in the anatase phase and the contribution of Ag nanoparticles did not cause any change in the obtained peaks. FTIR results confirmed that TiO_2_, Ag_2_S, and PANI were successfully synthesized. In EC, PC, and PEC activity experiments on methylene blue, PANI/Ag_2_S/TiO_2_ NT photoelectrode showed the best degradation activity with 13.775%, 37.71%, and 61.075% degradation rates, respectively.

## 4 Experimental Section

### Materials

Titanium, Foil, 2.0 mm Thickness, 99.7% Metals Basis, Aniline (C_6_H_7_N, ≥99.0%), Ortho‐Phosphoric acid (H_3_PO_4_, 85%), Sodium fluoride (NaF, 99.99%), Hydrochloric acid (HCl, 37%), Sodium sulfide (Na_2_S.9H_2_O, ≥98.0%), Ag/AgCl reference electrode, Sulfuric acid (H_2_SO_4_, 95‐97%) Ammonium persulfate (NH_4_)_2_S_2_O_8_, ≥98%), Silver nitrate (AgNO_3_, ≥99.0%) from Merck and Sigma Aldrich companies and methylene blue (C_16_H_18_C_l_N_3_S.xH_2_O) used in photoelectrocatalytic experiments. The dyestuff was obtained from Fluka company.

### Synthesis of TiO_2_ Nanotube Photoelectrodes

Ti foils with a thickness of 2.0 mm were cleaned with ultrasound in acetone, ethanol, and methanol for 10 min, respectively. Then, Ti Foils were anodized in 400 mL solution containing 0.14 m NaF and 0.5 m H_3_PO_4_ at 20 V for 1 hour. After the anodization process, the Ti foils were thoroughly washed with ionized water and dried. Finally, it was calcined at 500 °C for 3 hours. The anodization process will be carried out using a two‐electrode system (Ti foil as anode and Pt wire as cathode, with a distance of 3 cm between anode and cathode).

### Synthesis of Ag_2_S/TiO_2_ Nanotube Photoelectrodes

Ag_2_S/TiO_2_ NT was synthesized using the SILAR method. TiO_2_ NTs synthesized by anodization were dipped into 4 beakers respectively and the coating process was carried out. The coating process is as follows. The 1st beaker contains 0.01 m AgNO_3_, the 2nd beaker contains ionized water, the 3rd beaker contains 0.01 m Na_2_S and the 4th beaker contains ionized water.^[^ ^27^ ^]^ TiO_2_ NTs were immersed in an AgNO_3_ solution and cleaned in deionized water, then immersed in a Na_2_S solution and cleaned in deionized water. The coating process was repeated five times. To obtain the anatase TiO_2_ phase, Ag_2_S/TiO_2_ NTs were dried and calcined at 500 °C in air for 3 h.

### Synthesis of PANI/Ag_2_S/TiO_2_ Nanotube Photoelectrodes

PANI polymer was synthesized by the chemical oxidation method. 0.01 m aniline was mixed in ionized water in a beaker. On the other hand, 0.01 m APS was dissolved in ionized water in another beaker. After 15 min of mixing, the APS solution was added dropwise into the aniline solution. Then, the pH was adjusted to 2.3 with the help of HCl acid. Finally, the PANI polymer was coated on Ag_2_S/TiO_2_ NTs with the help of sol‐gel. During the coating process, the Ti foil was kept in the solution for 1 min and the coating process was repeated five times. Finally, it was air dried.

### Characterization of TiO_2_ NT Photoelectrodes

Morphological characteristics and elemental analysis of photoelectrodes were investigated using scanning electron microscopy (SEM‐EDS, JEOL, JSM‐7001F). Fourier transform infrared spectroscopy (FTIR, Bruker, TENSOR 27) was used to demonstrate the completion of the reaction in synthesizing TiO_2_ nanotubes modified with metal sulfide and polymer. The crystalline phases of the samples were determined by an X‐ray diffraction device (Rigaku, SmartLab) containing Cu‐Kβ radiation (λ = 1.3923).

### Electrochemical Measurements

Electrochemical impedance spectroscopy and potentiodynamic corrosion measurements were performed with a three‐electrode system connected to a Gamry potentiostat/galvanostat (model G‐300). Ti foil (1x1 cm) was used as the anode, Pt wire as the cathode, and Ag/AgCl as the reference. For measurements, three electrodes were immersed in 1 m H_2_SO_4_ solution.

### Photoelectrocatalytic Degradation of Methylene Blue

The photocatalytic, electrocatalytic, and photoelectrocatalytic activities of TiO_2_ NTs were tested in the degradation of methylene blue dyestuff in an aqueous solution. As experimental parameters, 400 mL solution volume, 20 ppm dye concentration, 25 °C ambient temperature, 254 nm (44W/m^2^) UV light source, and 0.6 V operating voltage were selected. The solution was stirred in the dark for 30 min to ensure adsorption‐desorption balance before the photoreaction started. O_2_ support was provided to the solution medium by an air pump and 0.05 m Na_2_SO_4_ was added. Degradation experiments were carried out using two electrode systems. The concentration of samples taken at certain time intervals was determined at 668 nm on a UV spectrophotometer (Optizen α).

## Conflict of Interest

The authors declare no conflict of interest.

## Data Availability

The data that support the findings of this study are available from the corresponding author upon reasonable request.
